# Energy-exergy and environ-economic (4E) analysis of heat storage-based single-slope solar stills integrated with solar air heater

**DOI:** 10.1371/journal.pone.0314036

**Published:** 2025-01-15

**Authors:** Sujit Kumar, Asim Ahmad, Kashif Irshad, Om Prakash, Rukaiya Kausher, S. M. Mozammil Hasnain, Prabhu Paramasivam, Abinet Gosaye Ayanie, Jayant Giri

**Affiliations:** 1 Department of Mechanical Engineering, Birla Institute of Technology, Ranchi, India; 2 Interdisciplinary Research Center for Sustainable Energy Systems (IRC-SES), King Fahd University of Petroleum and Minerals (KFUPM), Dhahran, Saudi Arabia; 3 Mechanical Engineering Department, King Fahd University of Petroleum and Minerals (KFUPM), Dhahran, Saudi Arabia; 4 Department of Civil and Environmental Engineering, Birla Institute of Technology, Mesra, Ranchi, India; 5 Faculty of Engineering & Technology, Department of Mechanical Engineering, Marwadi University Research Center, Marwadi University, Rajkot, Gujarat, India; 6 Department of Mechanical Engineering, Adama Science and Technology University, Adama, Ethiopia; 7 Department of Mechanical Engineering, Yeshwantrao Chavan College of Engineering, Nagpur, India; 8 Division of Research and Development, Lovely Professional University, Phagwara, India; 9 Chitkara University Institute of Engineering and Technology, Centre for Research Impact & Outcome, Chitkara University, Rajpura, Punjab, India; Agricultural Sciences and Natural Resources University of Khuzestan, ISLAMIC REPUBLIC OF IRAN

## Abstract

The energy-exergy and environ-economic (4E) analysis was conducted on a solar still with and without a hybrid thermal energy storage system (TESS) and a solar air heater. The proposed solar still was modified by integrating a rectangular aluminium box filled with paraffin wax and black gravel as the TESS and coupled with a solar air heater. Paraffin wax was selected due to its widespread availability and proven effectiveness in accelerating desalination, improving process uniformity, and maintaining optimal temperature levels. Throughout the experiments, meticulous data on mass loss, air velocity, and temperature were recorded for both conditions. The daily energy efficiency varies from 40.80% to 31.72%, showing a reduction rate with increased water depth. Estimates were made on the average exergy efficiency, losses, outflow, and inflow for the solar still. These were done for both setups. The analysis revealed that CO_2_ mitigation and credit were more favorable with the TESS. Furthermore, the Energy Payback Time (EPBT) for the hybrid heat storage-based single-slope solar still coupled with a solar air heater is 1.87 years. On the other hand, EPBT values for the hybrid heat storage single-slope solar still and the conventional single-slope solar still were 1.65 years and 0.95 years, respectively. Integrating a thermal energy storage system and solar air heater significantly improved the performance and sustainability of the solar still for desalination, making it a more efficient and environmentally friendly option for freshwater production.

## 1. Introduction

Environmental degradation accelerates with the passage of time. Global warming is rapidly melting glaciers, disrupting climate patterns and elevating average temperatures. Earth contains a vast supply of water but only a small fraction is suitable for human consumption, with much of this portion remaining largely inaccessible. Due to its critical importance to human survival, addressing the challenge of water scarcity has become increasingly vital. Water purification and distribution systems are often compromised during natural disasters or conflicts, leaving populations vulnerable. Moreover, underdeveloped regions lack adequate infrastructure for delivering clean water, exacerbating the crisis [1].

This highlights the critical need for autonomous, portable water purification devices. Solar stills emerge as a promising solution, capable of transforming brackish water into potable liquid without relying on traditional fuels. This environmentally friendly technology harnesses solar energy to drive a distillation process, effectively removing impurities and heavy metals. Although initial freshwater yields were relatively low, ongoing research has led to improved models with enhanced productivity [2]. A substantial body of literature and dedicated research efforts have been instrumental in advancing the development of these promising designs.

To enhance yield, researchers have modified traditional solar still design by focusing on increasing the evaporation rates of basin water. This improvement is achieved through a detailed analysis of heat transfer processes between the still and its environment [3–6]. Notable innovations include double-slope stepped solar stills with continuous water circulation, stepped solar stills, solar stillsincorporating phase change materials, hybrid solar stills, stills with thermal storage materials, photovoltaic-thermal integrated stills, and stills vertical ripple surfaces [7–10]. Numerous other inventive approaches have also been explored over the past four to five decades.

Joshia and Tiwari enhanced an active solar still by incorporating a heat exchanger and integrating it with a flat plate water collector and N-PVT system [11]. Experimental results demonstrated significantly improved performance compared to a traditional active single solar still, particularly at a water depth of 0.14 meters. Exergoeconomic, productivity, and enviroeconomic analyses confirmed the superiority of the active double slope solar still integrated with PVT (ADSSS/PVT). Heat transfer and operating temperature are primary determinants of still yield. Reflectors, solar collectors, and concentrators have been widely employed to augment solar still productivity [12]. Omara et al. experimentally investigated modified stepped solar stills with internal reflectors, achieving a productivity increase of up to 75% compared to standard stills [13]. Tanaka designed and tested a solar still with internal and external reflectors, reporting a 70–100% increase in daily yield during winter [14].

Omara et al. [15] utilized a modified stepped solar still equipped with internal reflectors, resulting in a 75% increase in productivity. Various types of concentrators have been attached to solar stills to increase productivity, and Tiwari and Suneja [16] conducted a numerical study on this topic. Chaochi et al. [17] equipped a solar desalination setup with a parabolic concentrator in their experimental study and formulated a theoretical model to derive the absorber temperature and distillate flow rate as functions of solar radiation.

The theoretical model exhibited an average relative inaccuracy of 42% in predicting flow rate. Gorjian et al. developed, constructed, and evaluated a standalone point-focus parabolic solar still for saltwater desalination [18]. The solar still achieved a maximum daily yield of 5.12 kg/m^2^/day and an efficiency of 36.7%. Researchers have proposed various configurations combining different solar stills with solar collectors. Arunkumar et al. constructed four non-concentrating and three concentrating solar stills, experimentally assessing their performance [19]. The compound parabolic collector coupled with the pyramid solar still demonstrated the highest productivity. This combination facilitated the formation of thin water films across the drum, accelerating water evaporation rates [20–25].

Energy and exergy analyses using the first and second laws of thermodynamics are employed for quantitative and qualitative assessments of energy systems. Exergy analysis helps identify and understand system inefficiencies, as well as determine their magnitude and locations within renewable energy systems [26–37]. However, exergy analysis has been found to be less common compared to energy analysis for renewable energy systems.

Dwivedi and Tiwari conducted an energy analysis of a solar still, revealing a 51% performance enhancement in active compared to passive systems [22,23]. Ranjan et al.’s energy and exergy analysis indicated negligible exergy inefficiencies relative to energy inefficiencies [24]. Tiwari et al. compared the thermal performance of active and passive solar stills through energy and exergy analysis [25]. Table 1 presents a comparison of various studies conducted in the field of solar still technology.

**Table 1:** Various studies conducted in the field of solar still.

Sr. No	Authors	Approach Taken	Key Findings
1	Punniakodi & Senthil [38]	Experimented with phase change materials (PCMs) in different container geometries	Increased PCM melting rate by up to 71%
2	Ahmed et al. [39]	Utilized a parabolic trough collector (PTC) with a tubular solar still (SS)	Achieved a 31.65% improvement in distillate output without additional production costs
3	Essa et al. [40]	Employed convex absorber surfaces and nanomaterials with tubular SS	Convex surface improved vaporization, boosting SS distillate yield
4	Serradj et al. [41]	Conducted experiments with baffles in single slope SS	Baffles enhanced natural convection, increasing distillate production
5	Subramanian et al. [42]	Investigated pyramid-shaped SS coupled with a flat plate collector (FPC)	Reduced glass-water gap and preheated basin water, achieving 50% higher distillate
6	Vigneswaran et al. [43]	Conducted exergy, energy, and economic analysis of SS using PCMs	Improved economic performance and reduced cost per liter (CPL) with PCM addition
7	Nien et al. [44]	Conducted an economic evaluation of SS with PCMs	Found that PCMs enhanced diurnal, nocturnal, and overall daily distillate output while reducing the energy payback period of SS
8	Present Study	Conducted an energy, exergy, environmental and economic evaluation of heat storage based hybrid SSSS.	The study highlights that optimizing water depth and integrating hybrid heat storage (Sensible, Latent, and SH+LH) with solar air heaters significantly enhances solar still efficiency, achieving up to 40.8%.

Punniakodi and Senthil experimented with phase change materials (PCMs) in different container geometries, achieving a notable increase in PCM melting rates by up to 71%. Ahmed et al. utilized a parabolic trough collector (PTC) with a tubular solar still, resulting in a 31.65% improvement in distillate output without additional production costs. Essa et al. enhanced tubular solar stills by employing convex absorber surfaces and nanomaterials, which improved vaporization and boosted distillate yield.

Serradj et al. incorporated baffles into single slope solar stills, enhancing natural convection and increasing distillate production. In contrast, Subramanian et al. investigated pyramid-shaped solar stills coupled with flat plate collectors, achieving a 50% increase in distillate output by reducing the glass-water gap and preheating the basin water. Vigneswaran et al. conducted an exergy, energy, and economic analysis of solar stills using PCMs, highlighting improvements in economic performance and reductions in cost per liter.

Nien et al. performed an economic evaluation of solar stills with PCMs, revealing enhancements in diurnal, nocturnal, and overall daily distillate output while decreasing the energy payback period. The present study expanded on this research by conducting a comprehensive evaluation of a heat storage-based hybrid single slope solar still. It demonstrated that optimizing water depth and integrating hybrid heat storage (Sensible, Latent, and SH+LH) with solar air heaters can significantly enhance solar still efficiency, achieving efficiencies of up to 40.8%. Collectively, these studies highlight the advancements in solar still technology and their potential to address water scarcity effectively.

Despite significant advancements in solar still technologies and numerous experimental studies focused on energy and exergy analysis, there is still a lack of comprehensive research investigating the combined effects of different thermal energy storage systems—sensible, latent, and hybrid—on the overall performance, sustainability, and exergy destruction of solar stills [26–30]. Furthermore, the integration of solar air heaters with solar stills for enhanced desalination efficiency, coupled with a thorough environ-economic analysis, remains underexplored [31–37].

This study addresses the above gaps by evaluating the energy-exergy and environ-economic aspects of various heat storage configurations and their impact on solar still productivity and environmental impact. The primary objective of this study is to perform a detailed energy, exergy, and environ-economic (4E) analysis of heat storage-based single-slope solar stills integrated with solar air heaters. Specifically, the study focuses on evaluating three different thermal energy storage configurations: (1) Sensible Heat Storage, (2) Latent Heat Storage using paraffin wax, and (3) a Hybrid Heat Storage system that combines both sensible and latent heat storage. These setups are tested at various water depths (3 cm, 6 cm, 9 cm, 12 cm, and 15 cm) to assess their influence on desalination efficiency and thermal performance. The investigation aims to comprehensively examine the energy efficiency, exergy efficiency, and exergy destruction across different components of the solar still, with a particular emphasis on the basin liner, which is identified as the component with the highest exergy destruction. This study also explores the environmental impact of the developed solar still systems, particularly in terms of CO_2_ mitigation, and evaluates the economic feasibility through key metrics like embodied energy, payback period, and Energy Payback Time (EPBT). Furthermore, the integration of the solar air heater with the solar still is analyzed to determine its impact on enhancing water evaporation rates and overall system productivity. The objective is to provide a thorough understanding of how different heat storage mechanisms and system configurations can improve both the energy and environmental sustainability of solar stills, making them a more efficient and practical solution for freshwater production through desalination.

## 2. Experimental setup and uncertainty analysis

**Fig 1:**
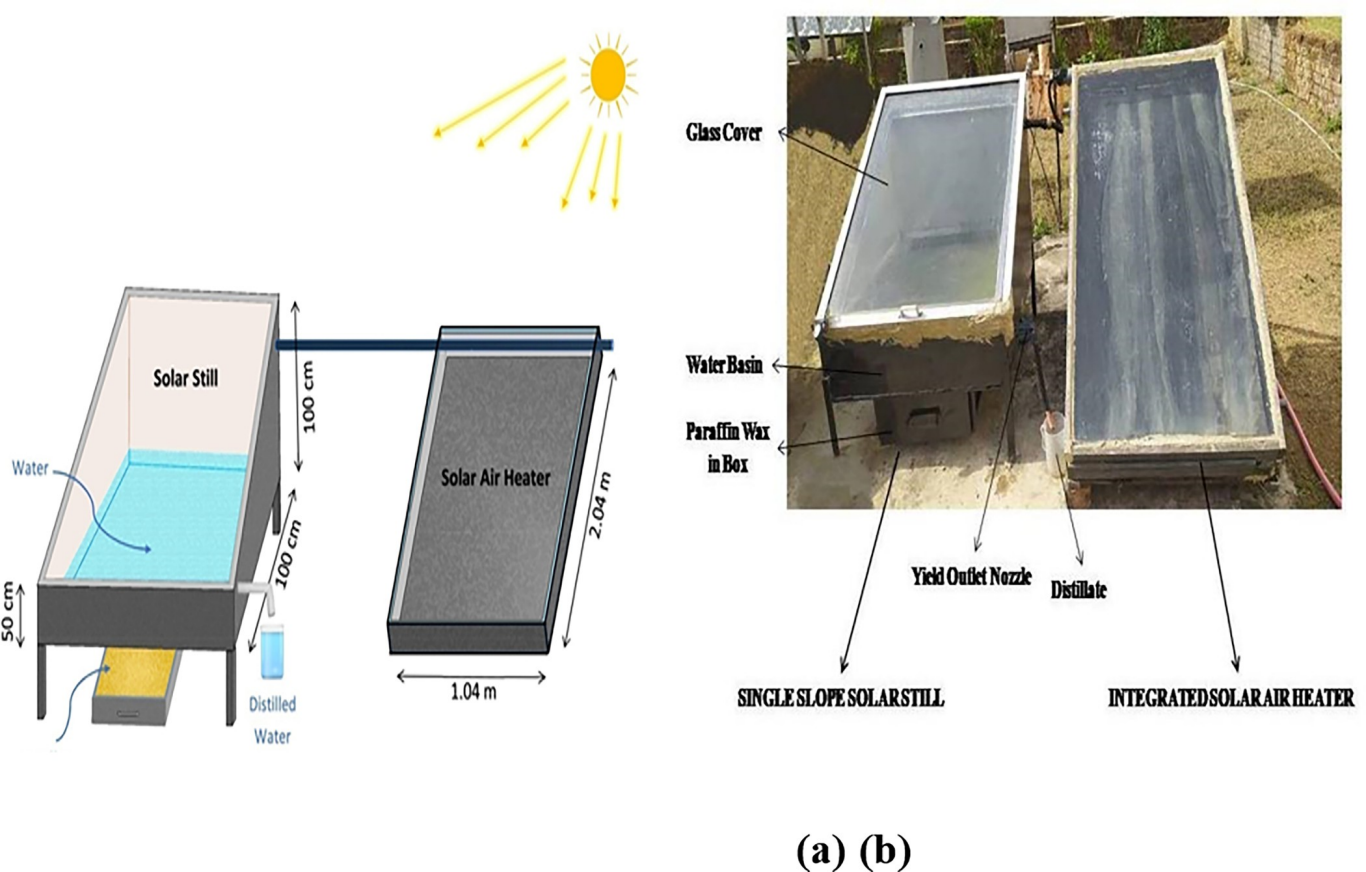
Single slope solar still with SAH (a) schematic view (b) real view.

For the experimental setup, three identical solar stills were developed and used as the base for each configuration, as shown in Fig 1. The first one was used as conventional still. The second one was enhanced by adding sensible (Black gravel) and latent heat storage (Paraffin wax), while the third was coupled with solar air heater. Black gravel has a thermal conductivity of 1.69 W/m·˚C, a density ranging from 2800 to 3000 kg/m^3^, and a specific heat capacity of 1230 kJ/kg·˚C. On the other hand, paraffin wax has a thermal conductivity of 1.69 W/m·˚C, with a density of 760 kg/m^3^ in solid form and 818 kg/m^3^ in liquid form. Its specific heat capacity is 2.95 kJ/kg·˚C at solid form and 2.51 kJ/kg·˚C at liquid form. Paraffin wax also has a latent heat of 226 kJ/kg and a melting temperature of 56°C. Welded iron sheets of 0.15 cm thickness was used to make the body of the solar still having dimension as (1 m length × 1 m width) with a basin area of 1 m^2^. Depth of high and low sided of the solar still were 45 cm and 16 cm respectively with inner surfaces painted to black. For minimizing the heat loss stills were properly insulated from all sides and upper surface was covered by glass with an inclination of 23.45° (latitude angle of Ranchi, India).

Experiment was conducted at BIT Mesra, Ranchi, India, from October 15 to November 25, 2020. Parameters such as hourly output, ambient temperature, wind speed, glass and hourly radiation, were measured on an hourly basis. Various instruments were used to ensure accurate measurements. An HTC Infrared Thermometer (MTX-2) with a range of -50°C to 550°C and a resolution of 0.1°C, along with an uncertainty of ±0.05%, was employed to measure surface temperatures. Wind speed, ambient temperature, and humidity were monitored using an HTC Digital Anemometer (AVM-06), which has a wind speed range of 0.80 to 30.00 m/s, an ambient temperature range of -10°C to 60°C, and a relative humidity range of 20% to 80%, with respective least counts of 0.01 m/s, 0.1°C, and 0.1%, and an uncertainty of ±3%. A Tenmars Solar Power Meter (TM-207) was used to measure solar irradiance, with a range of up to 2000 W/m^2^, a resolution of 0.1 W/m^2^, and an uncertainty of ±5%. These instruments provided precise and reliable data for the experimental analysis. Table 2 presents the uncertainty and experimental error of the various instruments, including their range and accuracy.

**Table 2:** Instruments with uncertainty analysis.

Instruments	Range of Instrument	Resolution of Instrument	Uncertainty (%)
HTC Infrared Thermometer (Digital) (MTX-2)	-50°C to 550° C	Least Count– 0.1°C	± 0.05
HTC Digital Anemometer with Temperature and Humidity Tester Meter (AVM-06)	0.80 to 30.00 m/s (Wind speed) -10°C to 60°C (Ambient temperature) 20 to 80% (Relative Humidity)	Least Count– 0.01 m/s (For Anemometer) Least Count– 0.1°C (For Ambient Temperature) & 0.1% (Relative Humidity)	± 3
Tenmars Solar Power Meter with Wire Sensor (TM-207)	2000W/m^2^	0.1 W/m^2^	± 5

## 3. Energy & exergy analysis

### 3.1 Energy analysis

The application of the energy balance principle was integral in assessing multiple components of the solar still, encompassing the glass cover, brackish water, and absorber plate. The primary objective of the energy analysis was to formulate a series of temperature-dependent equations, facilitating the calculation of temperatures for various components within the solar still. The energy balance equations for distinct elements within the system are articulated as follows [27]:

For glass cover:

$$\begin{array}{l} \mathrm{Energy}\;{\mathrm{output}}_{glass}=\mathrm{Energy}\;{\mathrm{input}}_{sky}\\ F_{g}+Q_{r,g-sky}+Q_{c,g-sky}=(H_{s}\times A_{g}\times AT_{g})+Q_{r,w-g}+Q_{e,w-g}+Q_{c,w-g}\\ F_{g}=(H_{s}\times A_{g}\times AT_{g})+Q_{r,w-g}+Q_{e,w-g}+Q_{c,w-g}-Q_{r,g-sky}-Q_{c,g-sky}\\ F_{g}={\left(m\times c_{p}\times (\frac{dT}{dt})\right)}_{g}\\ dT={\left([\frac{(H_{s}\times A_{g}\times AT_{g})+Q_{r,w-g}+Q_{e,w-g}+Q_{c,w-g}-Q_{r,g-sky}-Q_{c,g-sky}}{m\times c_{p}}]dt\right)}_{g} \end{array}$$ (1)

For water:

$$\begin{array}{l} \mathrm{Energy}\;{\mathrm{output}}_{water}=\mathrm{Energy}\;{\mathrm{input}}_{glass}\\ F_{w}+Q_{r,w-g}+Q_{c,w-g}+Q_{e,w-g}=(H_{s}\times A_{w}\times AT_{w})+Q_{c,b-w}\\ F_{w}=(H_{s}\times A_{w}\times AT_{w})+Q_{c,b-w}-Q_{r,w-g}-Q_{c,w-g}-Q_{e,w-g}\\ F_{w}={\left(m\times c_{p}\times (\frac{dT}{dt})\right)}_{w}\\ dT={\left([\frac{(H_{s}\times A_{w}\times AT_{w})+Q_{c,b-w}-Q_{r,w-g}-Q_{c,w-g}-Q_{e,w-g}}{m\times c_{p}}]dt\right)}_{w} \end{array}$$ (2)

For basin:

$$\begin{array}{l} \mathrm{Energy}\;{\mathrm{output}}_{ba\sin}=\mathrm{Energy}\;{\mathrm{input}}_{water}\\ F_{b}+Q_{c,b-w}+Q_{loss}=(H_{s}\times A_{b}\times AT_{b})\\ F_{b}=(H_{s}\times A_{b}\times AT_{b})-Q_{c,b-w}-Q_{loss}\\ F_{b}={\left(m\times c_{p}\times (\frac{dT}{dt})\right)}_{b}\\ dT={\left([\frac{(H_{s}\times A_{b}\times AT_{b})-Q_{c,b-w}-Q_{loss}}{m\times c_{p}}]dt\right)}_{b} \end{array}$$ (3)

Energy efficiency can be evaluated as [1]:

$$\eta_{th}=\frac{\sum \dot{m_{ew}h_{fg}}}{3600A_{p}\sum I(t)}$$ (4)

Where, m_ew_ is th amount of water produced and it can be evaluated as:

$$m_{ew}=\frac{h_{ew}A_{w}(T_{w}-T_{g})\times 3600}{h_{fg}}$$ (5)

### 3.2 Exergy analysis

Exergy analysis is derived from the second law of thermodynamics and acts as an indicator of energy’s ability to convert into work. Exergy can be explained as the maximum work a system can produce when it approaches thermodynamic equilibrium in a particular environment. The general equation for the exergy balance is given below [1]:

$$\sum {\dot{E}}_{x,i}-\sum {\dot{E}}_{x,o}=\sum {\dot{E}}_{x,d}$$ (6)

The solar irradiance exergy is used to estimate the exergy input to the solar still as shown in below [1]:

$$\sum {\dot{E}}_{x,i}=\sum {\dot{E}}_{x,s}=A_{b}I_{t}[1-\frac{4}{3}(\frac{T_{ambt}+273}{T_{S}})+\frac{1}{3}{\left(\frac{T_{ambt}+273}{T_{S}}\right)}^{4}]$$ (7)

Where,

T_s_ = 6000 K temperature of sun,

E_x,s_ = input of exergy to the solar still from solar insulation,

A_b_ = effective still basin area in m^2^,

And the accumulated incident solar irradiance on solar still is measured in W/m^2^. For the still used in this work, Eq 8 shows the exergy output of product which here is distil water [1].


$${\dot{E}}_{x,o}={\dot{E}}_{x,ev}=\frac{m_{ew}\lambda_{lt}}{3600}[1-(\frac{T_{ambt}+273}{T_{wr}+273})]$$ (8)

Where E_x,ev_ is exergy due to evaporation and λlt is the latent heat of evaporation.

$$\lambda_{lt}=3.16(10^{6}-761.6\times T_{i}),T_{i}>70$$

$$\lambda_{lt}=2.49(10^{6}-947.7\times T_{i}+0.13\times {T_{i}}^{2}-0.0047\times {T_{i}}^{3}),T_{i}<70$$

Where,

$$T_{i}=\frac{T_{wr}+T_{gl}}{2}$$ (9)

The exergy efficiency is calculated as the difference between the input and output exergy, and it is written as [1]

$$\eta_{ex}=\frac{{\dot{E}}_{x,ev}}{{\dot{E}}_{x,in}}$$ (10)

## 4. Environomical analysis

The Environomical analysis has been estimated for the heat storage-based single-slope solar stills, exploring their integration with solar air heaters. The experiments encompass three distinct setups involving Sensible Heat Storage, Latent Heat Storage, and a Hybrid Heat Storage system combining both Sensible and Latent Heat Storage.

### 4.1 Embodied energy

Embodied energy is defined as the total energy required for producing any product or service [28].

### 4.2 Energy payback time (EPBT)

The energy payback period is the required time to recover embodied energy of the product. It is determined as Eq 11 [29]:

$$EPBT=\frac{E_{emd}}{AE_{output}}$$ (11)

Where, *E*_*emd*_ is Embodied energy and *AE*_*output*_ is annual energy output.

Therefore, Energy payback time relies on embodied energy and annual energy output.

### 4.3 CO_2_ emission

The emission of average CO_2_ for Coal generated electricity as suggested by Prakash et al., approximately equivalent to 0.98kg of CO_2_/kWh [28]. The lifetime of the developed set up was found to be 10 years [30]. The CO_2_ emission per year, thus, can be calculated as Eq 12

$$CO_{2}\;\mathrm{Emission}\;\mathrm{per}\;\mathrm{year}=\frac{E_{emd}\times 0.98}{L}$$ (12)

Where, *L* is the lifetime of the developed system.

### 4.4 Cost analysis

The investment payback period of the developed setup is being calculated as:

$$N=\frac{\ln(\frac{CF}{CF-(AFC\times i)})}{\ln(1+i)}$$ (13)

Where, CF (Cash Flow) = Yearly yield * Selling price of distillate.

### 4.5 Carbon mitigation & earned carbon credit

To measure climate change potential, carbon dioxide mitigation is applied as a key metric. This allows for convenient comparison with other power production systems, as net CO₂ mitigation is measured per kilowatt-hour. Carbon credits are defined as a crucial component of national and international emissions trading schemes, which are implemented to mitigate global warming[31–34]. Minimizing greenhouse gas emissions on a commercial scale is achieved by capping total annual emissions while allowing for compensation in cases of shortfall in assigned greenhouse gas mitigation targets [35–37]. At current prices, carbon credits can be bought and sold in international markets or within businesses, making them usable in financial carbon reduction schemes [28]. The daily thermal output, daily thermal input, and annual thermal output energy can be evaluated using Eqs 14 to 16:

$$E_{an}=\mathrm{Daily}\;\mathrm{thermal}\;\mathrm{output}\;\mathrm{energy}\;\mathrm{of}\;\mathrm{still}(E_{d})\times N_{d}$$ (14)

$$E_{d}=\frac{M_{y}\times L_{ent}}{3.6\times 10^{6}}$$ (15)

$${\mathrm{Daily}\;\mathrm{input}\;\mathrm{energy}=I}_{g}\times N_{h}\times A_{c}\times 10^{-3}\mathrm{kWh}$$ (16)

Where, *M*_*y*_ is yearly distillate (kg/yr), *L*_*ent*_ is latent heat of evaporation, N_d_ is total number of sunshine days in a year i.e 300 days, E_an_ is annual thermal output energy, A_c_ is area of solar collector and N_h_ is the number of sunshine hours per day.

Coal based power is 0.98kg of CO_2_/kWh, due to mean CO_2_ equivalent intensity, so the amount of CO_2_ mitigation of the system would be as per Eqs 17 and 18.


$${\mathrm{Lifetime}\;\mathrm{mitigation}\;\mathrm{of}\;\mathrm{CO}}_{2}(kg)={\mathrm{Total}\;\mathrm{CO}}_{2}\;\mathrm{mitigation}-{\mathrm{Total}\;\mathrm{CO}}_{2}\;\mathrm{emission}$$ (17)

$${\mathrm{Lifetime}\;\mathrm{mitigation}\;\mathrm{of}\;\mathrm{CO}}_{2}(kg)=[(E_{an}\times n)-E_{m}]\times 2.01$$ (18)

Where, E_m_ is embodied energy in kWh. and n denotes the lifespan of the developed set up, which is 10 years. The earned carbon credit was found as per Eq 19

$${\mathrm{Earned}\;\mathrm{carbon}\;\mathrm{credit}=\mathrm{net}\;\mathrm{mitigation}\;\mathrm{of}\;\mathrm{CO}}_{2}\;\mathrm{in}\;\mathrm{lifetime}(\mathrm{tone})\times D$$ (19)

Here, the cost of carbon credit is denoted by D which varies from $5-20/ton of the CO_2_ mitigation.

## 5. Result & discussion

### 5.1 Comprehensive analysis of energy and exergy efficiency

A comprehensive examination of the energy and exergy analysis of a single slope solar still was conducted, considering varying water depths. The efficiency of both energy and exergy is intricately linked to meteorological factors, particularly the solar radiation within the local climate conditions. Solar radiation fluctuates throughout the experimental period, ranging from 800 to 1380 W/m^2^. To systematically explore the influence of water depths on energy and exergy efficiency, the experiments were designed to maintain a consistent total daily solar intensity while altering the water depths.

**Fig 2:**
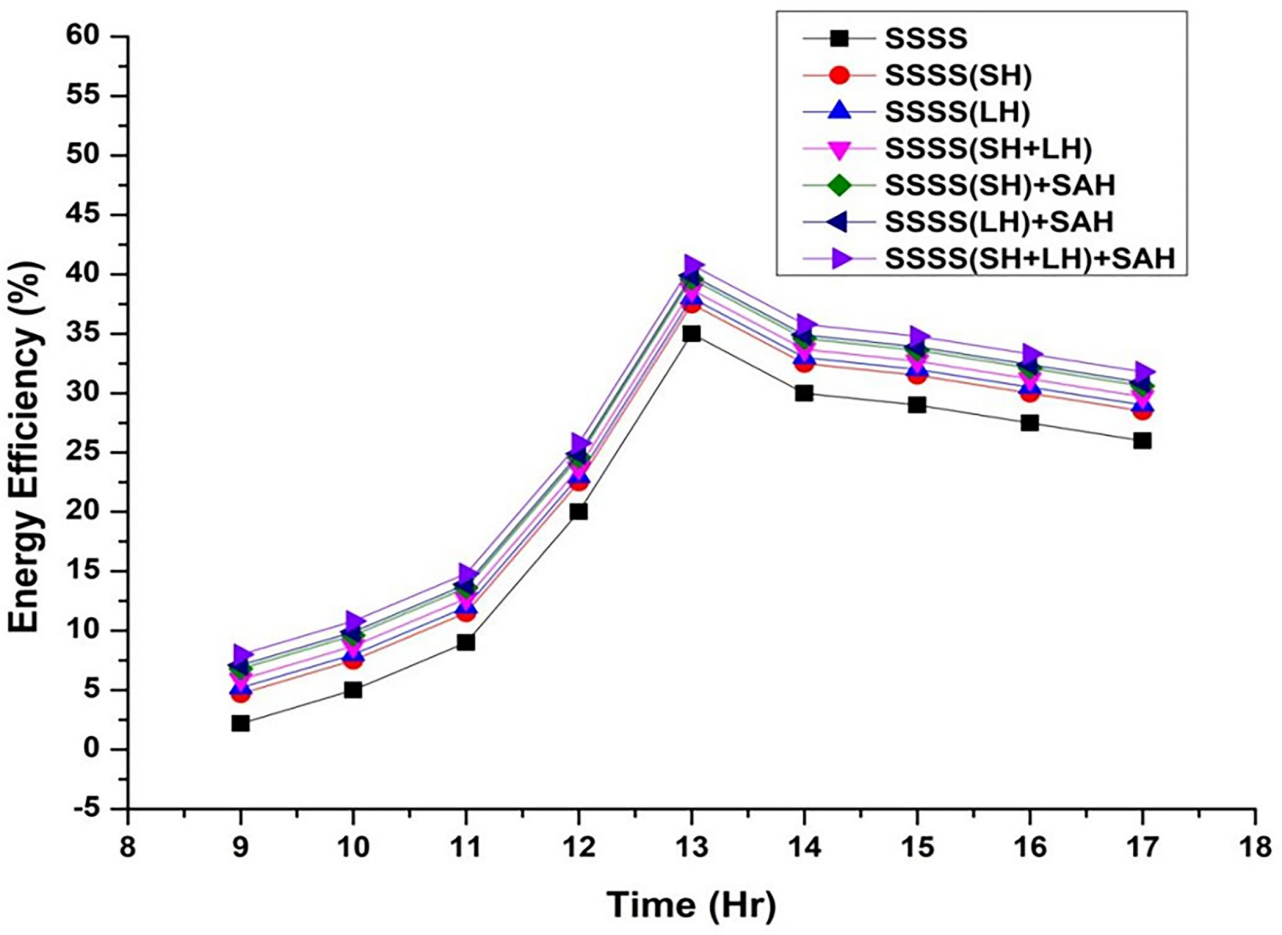
Variation of energy efficiency with time at 3 cm depth.

**Fig 3:**
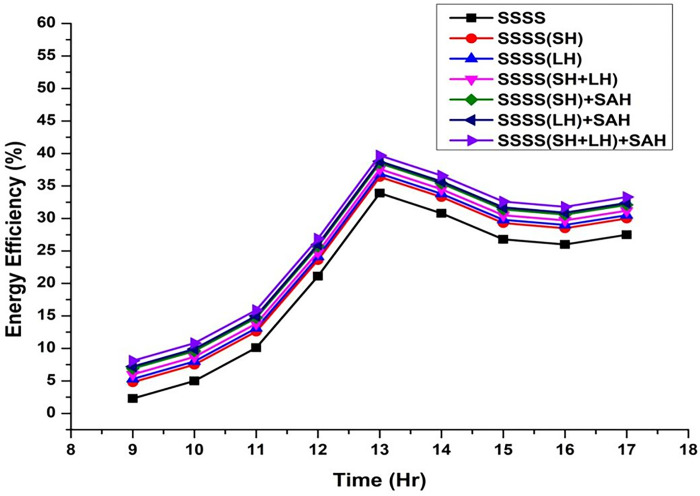
Variation of energy efficiency with time at 6 cm depth.

**Fig 4:**
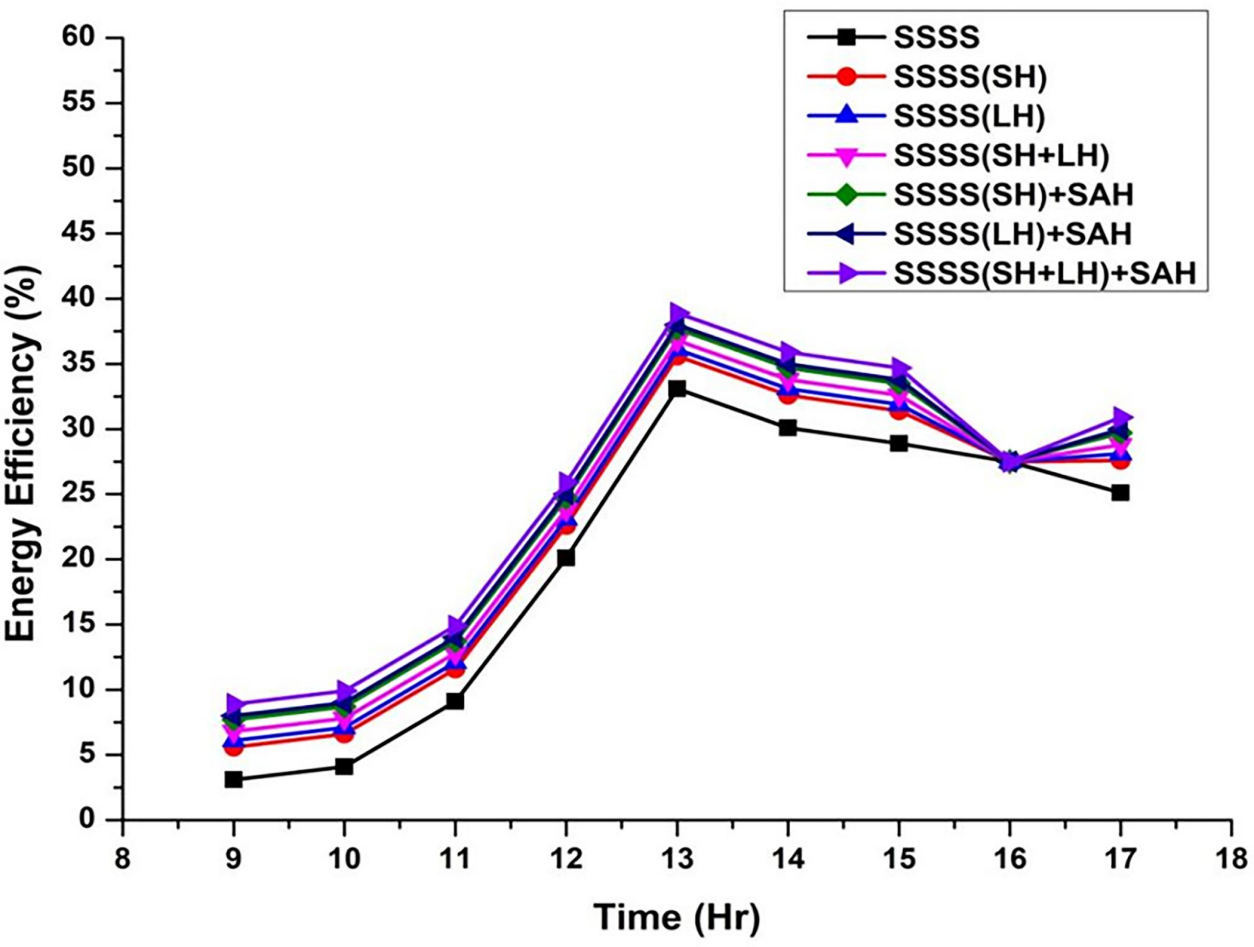
Variation of energy efficiency with time at 9 cm depth.

**Fig 5:**
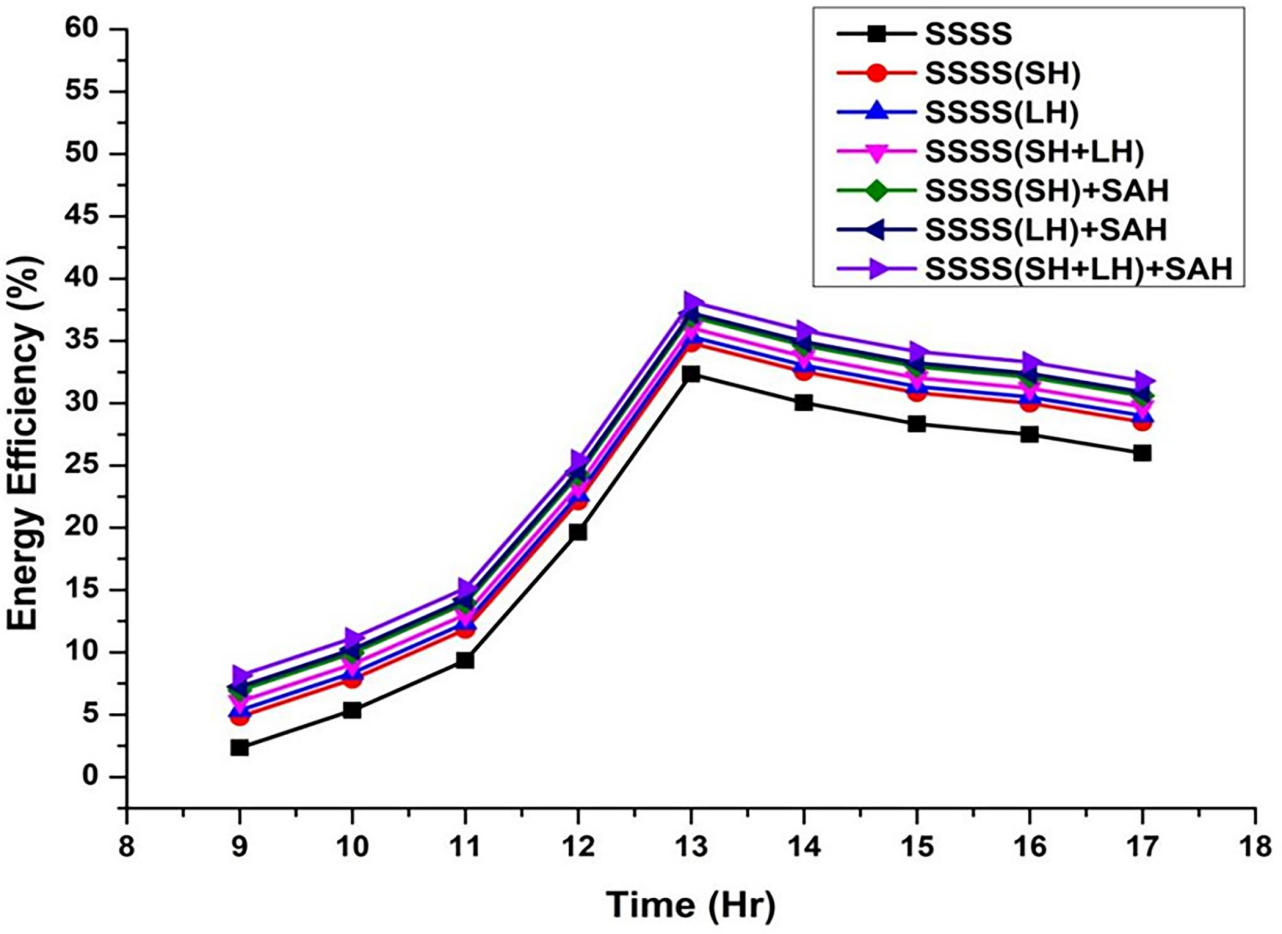
Variation of energy efficiency with time at 12 cm depth.

**Fig 6:**
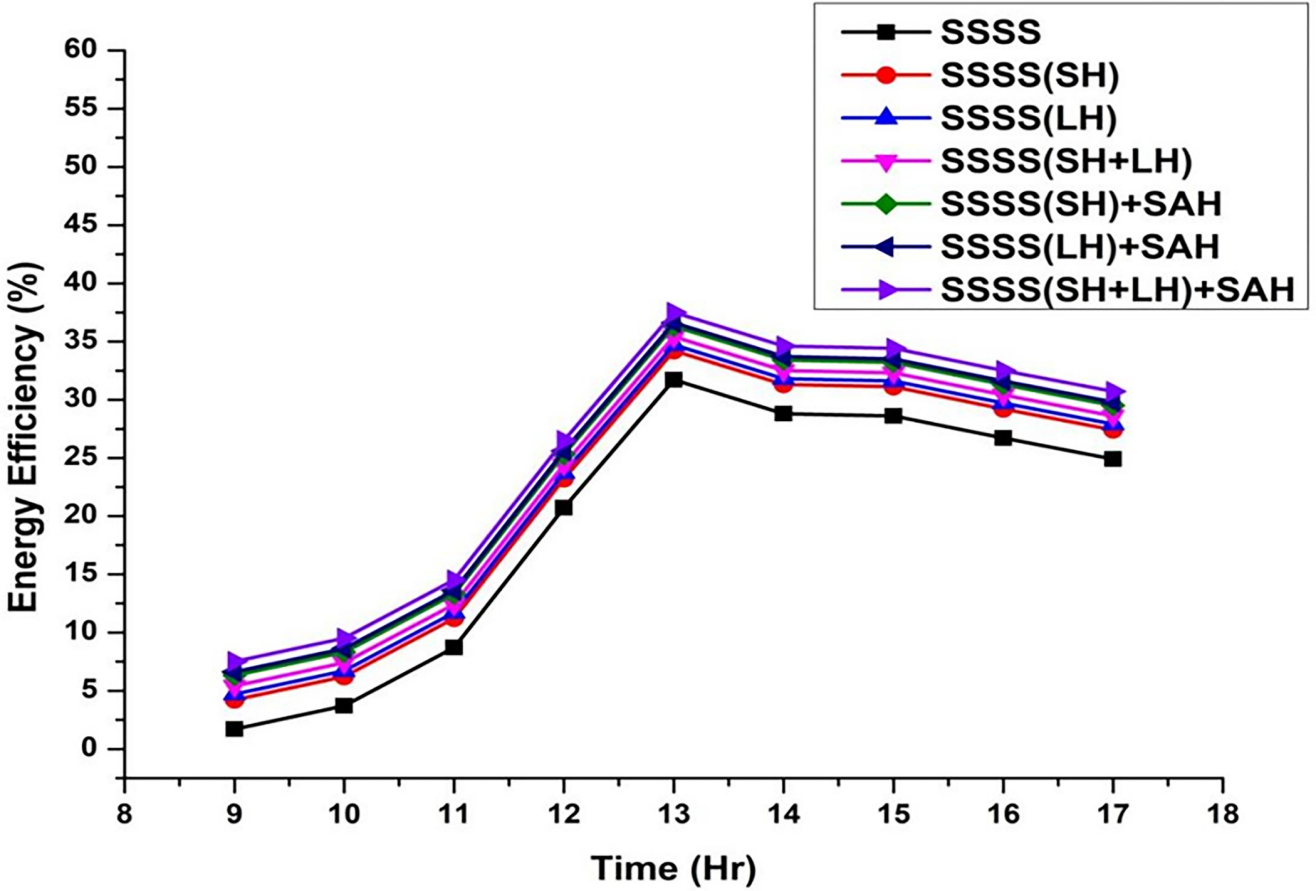
Variation of energy efficiency with time at 15 cm depth.

Throughout the experimental phase, three specific setups were implemented, incorporating Sensible Heat Storage, Latent Heat Storage, and a Hybrid Heat Storage system that integrates both Sensible and Latent Heat Storage. To gauge the effects of these configurations on system performance, paraffin wax and black-painted gravel were utilized as effective thermal storage materials at varying depths (3 cm, 6 cm, 9 cm, 12 cm, and 15 cm). This approach enabled a detailed investigation into the impact of different water depths on energy, exergy efficiency, and irreversibility associated with various components of the solar still. The variation of energy efficiency is shown from Fig 2–6.

The efficiencies of the Single Slope Solar Still (SSSS) were investigated across various water depths, revealing insightful trends in the system’s performance. The efficiency of the solar still decreased with increasing water depth. At a depth of 3 cm, the SSSS demonstrated an efficiency of 35%, while at 15 cm, the efficiency declined to 31.72%. This inverse relationship between efficiency and water depth underscores the importance of considering optimal depth parameters for the effective operation of single slope solar stills.

At a water depth of 3 cm, the SSSS exhibits an efficiency of 35%, while the addition of Sensible Heat (SH), Latent Heat (LH), and their combination (SH+LH) results in incremental efficiency improvements. Specifically, SSSS(SH) shows an efficiency of 37.5%, SSSS(LH) achieves 38%, and SSSS(SH+LH) attains an efficiency of 38.7%. The integration of Solar Air Heater (SAH) further enhances these efficiencies, with SSSS(SH)+SAH reaching 39.6%, SSSS(LH)+SAH achieving 39.9%, and SSSS(SH+LH)+SAH demonstrating the highest efficiency at 40.8%. These findings emphasize the positive impact of heat storage mechanisms and solar air heaters on the overall efficiency of the single slope solar still at a specific water depth.

Table 3 compares energy efficiency under different water depths and heat storage conditions. Observed daily energy efficiency in experiments ranges from 31.72% to 40.80%, decreasing with decreasing water depth (from 15 mm to 3 mm). Dunkle’s model predicts a maximum daily efficiency of 44.47%, while Clark’s model predicts a minimum of 30.13%. Both thermal models exhibit a similar trend, with increasing then decreasing daily energy efficiency. However, while experiments show a consistent decrease in efficiency with increasing water depth, the other models predict a fluctuating pattern of daily efficiency for higher water depths.

**Table 3:** Comparison of experimental energy efficiency with other models at different depth.

Depth	Experimental						
	SSSS	SSSS (SH)	SSSS (LH)	SSSS (SH+LH)	SSSS (SH)+SAH	SSSS (LH)+SAH	SSSS (SH+LH)+SAH
**3**	35%	37.5%	38%	38.7%	39.6%	39.9%	40.8%
**6**	33.9%	36.4%	36.9%	37.6%	38.5%	38.8%	39.7%
**9**	33.1%	35.6%	36.1%	36.8%	37.7%	38%	38.9%
**12**	32.34%	34.84%	35.34%	36.04%	36.94%	37.24%	38.14%
**15**	31.72%	34.22%	34.72%	35.42%	36.32%	36.62%	37.52%
**Depth**	**Dunkle Model**						
**3**	38.15%	40.87%	41.42%	42.18%	43.16%	43.49%	44.47%
**6**	36.95%	39.67%	40.22%	40.98%	41.96%	42.29%	43.27%
**9**	36.07%	38.80%	39.34%	40.11%	41.09%	41.42%	42.40%
**12**	35.25%	37.97%	38.52%	39.28%	40.26%	40.59%	41.57%
**15**	34.57%	37.29%	37.84%	38.60%	39.58%	39.91%	40.89%
**Depth**	**Clark Model**						
**3**	33.25%	35.62%	36.1%	36.76%	37.62%	37.90%	38.76%
**6**	32.20%	34.58%	35.05%	35.72%	36.57%	36.86%	37.71%
**9**	31.44%	33.82%	34.29%	34.96%	35.81%	36.1%	36.95%
**12**	30.72%	33.09%	33.57%	34.23%	35.09%	35.37%	36.23%
**15**	30.13%	32.50%	32.98%	33.64%	34.50%	34.78%	35.64%

The study also compared the experimental results of solar still systems with predictions made by Dunkle’s and Clark’s models to validate their performance. Dunkle’s model, based on heat and mass transfer relationships, provides a reasonable estimation of energy efficiency, particularly at lower water depths. It predicted a maximum daily energy efficiency of 38.13%, whereas the experimental efficiency varied from 19.21% to 30.97%. While Dunkle’s model showed a consistent trend of decreasing efficiency with increasing water depth, it tended to overestimate efficiency at higher depths.

In contrast, Clark’s model, which estimates evaporative heat transfer rates and is applicable under higher operating temperatures, predicted a maximum daily energy efficiency of 19.06%. However, this model often underestimated the experimental results, especially at lower water depths, highlighting its limitations in accurately predicting performance under varying conditions. Both models reveal discrepancies when compared to the experimental findings, indicating the need for careful validation against real-world data. Ultimately, while Dunkle’s model aligns well with lower temperature scenarios and shallow water depths, Clark’s model is better suited for higher temperatures and larger distances between the water surface and the glass cover. The study underscores the importance of experimental validation to enhance the applicability of theoretical models in solar still performance analysis.

The recorded data provides valuable insights into the impact of water depth on the efficiency of the SSSS. Such findings are essential for designing and optimizing the performance of solar stills, offering practical guidance for maximizing efficiency in real-world applications. The systematic examination of these efficiency variations contributes to the broader understanding of factors influencing the efficacy of single slope solar stills, thereby informing future advancements in solar desalination technologies.

Exergy analysis is instrumental in evaluating the efficiency of a system, primarily aiming to characterize and identify the sources of exergy losses within its components. The variation in the rate of exergy destruction for various components of the solar still reveals valuable insights.

The analysis reveals a direct correlation between exergy destruction rates in system components and the levels of incident solar insolation. Notably, irreversibility is found to be significantly higher in the absorber plate compared to the exergy destruction occurring in the glass cover and saline water. This phenomenon is primarily due to the temperature gradient between the sun and the basin plate, as well as the dissipation of exergy from the absorber plate into the atmosphere.

Reported maximum exergy destruction rates are 717 W/m^2^ for the absorber plate, 61.1 W/m^2^ for the glass cover, and 50.2 W/m^2^ for the saline water. Consequently, the overall exergy destruction rate for the system is calculated to be 828 W/m^2^, with the absorber plate contributing to 86% of the total irreversibility.

The analysis indicates that evaporation exergy and still efficiency increase with higher water temperatures. Various parameters, such as the temperature of the glass surface and atmospheric temperature, also impact exergy and efficiency. The influence of solar irradiation results in relatively high exergy, although the same does not hold true for the exergy efficiency of a solar collector.

In summary, a comprehensive overview of daily exergy output and exergy efficiency is provided in Tables 4–18. The findings underscore the significance of understanding and addressing exergy losses in different components to enhance the overall efficiency of the solar still system.

**Table 4:** Exergy analysis of developed setup with sensible heat storage integration at 3 cm depth.

Time	Depth	SSSS			SSSS (SH)			SSSS (SH + SAH)		
		Ex_in_ (kJ/m^2^)	Ex_out_ (kJ/m^2^)	n_eff_ (%)	Ex_in_ (kJ/m^2^)	Ex_out_ (kJ/m^2^)	n_eff_ (%)	Ex_in_ (kJ/m^2^)	Ex_out_ (kJ/m^2^)	n_eff_ (%)
9	3 cm	915.75	0	0	726.582	0	0	779.513	0	0
10		1130	0.30	0.027	823.188	0.243	0.028	990.198	0.692	0.066
11		1170.74	0.35	0.03	946.318	0.371	0.037	1056.484	2.450	0.220
12		1221.48	1.24	0.10	1070.185	1.517	0.134	1095.702	4.663	0.404
13		1258.44	3.31	0.26	1082.625	5.095	0.506	1103.177	11.096	0.955
14		1002.19	4.80	0.48	892.720	9.413	0.999	917.330	13.857	1.435
15		801.77	7.50	0.93	459.640	11.829	2.445	723.925	17.007	2.233
16		708.70	8.14	1.15	318.250	12.518	3.736	431.556	21.618	4.738
17		606.22	10.37	1.71	106.128	14.139	12.656	87.951	15.248	15.470

**Table 5:** Exergy analysis of developed setup with sensible heat storage integration at 6 cm depth.

Time	Depth	SSSS			SSSS (SH)			SSSS (SH + SAH)		
		Ex_in_ (kJ/m^2^)	Ex_out_ (kJ/m^2^)	n_eff_ (%)	Ex_in_ (kJ/m^2^)	Ex_out_ (kJ/m^2^)	n_eff_ (%)	Ex_in_ (kJ/m^2^)	Ex_out_ (kJ/m^2^)	n_eff_ (%)
9	6 cm	915.62	0	0	664.209	0	0	770.405	0	0
10		726.53	0.17	0.024	1055.713	0.632	0.056	733.797	1.002	0.129
11		1086.76	0.42	0.039	720.793	0.453	0.059	1170.918	2.263	0.183
12		1132.07	1.43	0.12	962.018	2.137	0.210	1175.199	3.315	0.267
13		1114.50	1.76	0.15	897.247	2.236	0.236	1187.946	4.800	0.384
14		946.44	3.50	0.37	853.210	3.391	0.377	938.018	4.907	0.496
15		828.39	4.96	0.60	758.955	5.414	0.678	726.600	5.270	0.689
16		726.18	6.24	0.86	602.561	6.490	1.023	554.548	11.498	1.969
17		558.91	6.59	1.18	460.836	7.286	1.502	485.483	12.667	2.478

**Table 6:** Exergy analysis of developed setup with sensible heat storage integration at 9 cm depth.

Time	Depth	SSSS			SSSS (SH)			SSSS (SH + SAH)		
		Ex_in_ (kJ/m^2^)	Ex_out_ (kJ/m^2^)	n_eff_ (%)	Ex_in_ (kJ/m^2^)	Ex_out_ (kJ/m^2^)	n_eff_ (%)	Ex_in_ (kJ/m^2^)	Ex_out_ (kJ/m^2^)	n_eff_ (%)
9	9 cm	905.22	0	0	451.763	0	0	610.736	0	0
10		874.89	0.09	0.01	1054.557	0.673	0.060	595.202	0.045	0.072
11		1004.70	0.21	0.02	646.389	0.034	0.050	1072.204	1.076	0.095
12		1050.73	0.35	0.03	866.072	0.642	0.070	1107.729	3.151	0.270
13		1142.27	1.14	0.09	928.674	1.975	0.201	1022.687	4.278	0.397
14		1022.63	1.81	0.17	734.270	2.873	0.371	863.369	4.037	0.444
15		741.37	3.46	0.46	685.777	3.898	0.540	672.904	4.106	0.579
16		652.16	4.38	0.67	530.267	4.018	0.720	584.407	5.082	0.826
17		539.84	5.20	0.96	557.909	5.944	1.012	487.265	7.135	1.391

**Table 7:** Exergy analysis of developed setup with sensible heat storage integration at 12 cm depth.

Time	Depth	SSSS			SSSS (SH)			SSSS (SH + SAH)		
		Ex_in_ (kJ/m^2^)	Ex_out_ (kJ/m^2^)	n_eff_ (%)	Ex_in_ (kJ/m^2^)	Ex_out_ (kJ/m^2^)	n_eff_ (%)	Ex_in_ (kJ/m^2^)	Ex_out_ (kJ/m^2^)	n_eff_ (%)
9	12 cm	822.00	0	0	655.547	0	0	265.817	0	0
10		875.93	0.10	0.011	918.305	0.19	0.019	946.815	0.267	0.026
11		1123.90	0.20	0.018	850.046	0.398	0.044	984.352	0.736	0.070
12		1033.44	0.12	0.011	1106.055	0.740	0.063	981.819	0.887	0.085
13		1097.02	1.70	0.15	1179.532	2.141	0.172	999.073	2.075	0.197
14		1006.56	2.52	0.25	907.282	2.460	0.257	857.201	2.565	0.284
15		810.99	2.57	0.31	626.924	2.795	0.424	706.710	3.393	0.456
16		641.67	2.44	0.38	673.209	2.718	0.383	531.474	2.942	0.525
17		530.19	2.75	0.51	515.445	3.600	0.663	611.640	5.381	0.835

**Table 8:** Exergy analysis of developed setup with sensible heat storage integration at 15 cm depth.

Time	Depth	SSSS			SSSS (SH)			SSSS (SH + SAH)		
		Ex_in_ (kJ/m^2^)	Ex_out_ (kJ/m^2^)	n_eff_ (%)	Ex_in_ (kJ/m^2^)	Ex_out_ (kJ/m^2^)	n_eff_ (%)	Ex_in_ (kJ/m^2^)	Ex_out_ (kJ/m^2^)	n_eff_ (%)
9	15 cm	830.79	0	0	682.197	0	0	850.193	0	0
10		1033.49	0.01	0.013	801.384	0.167	0.019	979.451	0.038	0.037
11		1134.62	0.16	0.014	911.703	0.204	0.021	1103.204	0.835	0.071
12		1042.92	0.50	0.048	1112.367	0.540	0.046	1116.431	3.071	0.262
13		1280.53	1.00	0.078	1140.344	0.921	0.076	1138.984	8.115	0.677
14		939.93	0.81	0.086	1112.077	2.249	0.192	1057.068	12.581	1.129
15		726.66	1.31	0.181	714.254	3.737	0.497	889.818	14.630	1.562
16		605.15	1.99	0.329	698.926	3.824	0.520	637.511	11.859	1.767
17		582.19	2.70	0.464	556.127	2.965	0.534	602.432	13.574	2.140

**Table 9:** Exergy analysis of developed setup with latent heat storage integration at 3 cm depth.

Time	Depth	SSSS			SSSS (LH)			SSSS (LH + SAH)		
		Ex_in_ (kJ/m^2^)	Ex_out_ (kJ/m^2^)	n_eff_ (%)	Ex_in_ (kJ/m^2^)	Ex_out_ (kJ/m^2^)	n_eff_ (%)	Ex_in_ (kJ/m^2^)	Ex_out_ (kJ/m^2^)	n_eff_ (%)
9	3 cm	915.75	0	0	764.824	0	0	820.54	0	0
10		1130	0.298	0.026	866.514	0.256	0.029	1042.26	0.729	0.069
11		1170.738	0.35	0.029	996.125	0.391	0.039	1112.09	2.579	0.231
12		1221.475	1.24	0.101	1126.512	1.597	0.141	1153.37	4.909	0.425
13		1258.443	3.307	0.262	1139.606	6.416	0.563	1161.24	11.68	1.005
14		1002.189	4.793	0.478	939.706	9.909	1.054	965.61	14.586	1.510
15		801.77	7.502	0.935	483.832	12.452	2.573	761.5	17.902	2.350
16		708.693	8.137	1.148	335	13.177	3.933	454.27	22.756	4.987
17		606.22	10.366	1.709	111.714	14.884	13.323	92.58	16.051	17.337

**Table 10:** Exergy analysis of developed setup with latent heat storage integration at 6 cm depth.

Time	Depth	SSSS			SSSS (LH)			SSSS (LH + SAH)		
		Ex_in_ (kJ/m^2^)	Ex_out_ (kJ/m^2^)	n_eff_ (%)	Ex_in_ (kJ/m^2^)	Ex_out_ (kJ/m^2^)	n_eff_ (%)	Ex_in_ (kJ/m^2^)	Ex_out_ (kJ/m^2^)	n_eff_ (%)
9	6 cm	915.626	0	0	699.168	0	0	810.953	0	0
10		726.528	0.173	0.023	1111.277	0.666	0.059	772.418	1.055	0.136
11		1086.761	0.427	0.039	758.73	0.477	0.062	1232.56	2.382	0.193
12		1132.074	1.432	0.126	1013.704	2.25	0.221	1237.052	3.49	0.282
13		1114.5	1.763	0.158	944.471	2.354	0.249	1249.418	5.053	0.404
14		946.445	3.5	0.369	898.117	3.57	0.397	988.44	5.165	0.522
15		828.394	4.964	0.599	797.853	5.699	0.714	764.843	5.548	0.725
16		726.181	6.248	0.860	634.275	6.832	1.077	583.735	12.104	2.073
17		558.908	6.592	1.179	485.091	7.67	1.581	511.035	13.334	2.609

**Table 11:** Exergy analysis of developed setup with latent heat storage integration at 9 cm depth.

Time	Depth	SSSS			SSSS (LH)			SSSS (LH + SAH)		
		Ex_in_ (kJ/m^2^)	Ex_out_ (kJ/m^2^)	n_eff_ (%)	Ex_in_ (kJ/m^2^)	Ex_out_ (kJ/m^2^)	n_eff_ (%)	Ex_in_ (kJ/m^2^)	Ex_out_ (kJ/m^2^)	n_eff_ (%)
9	9 cm	905.227	0	0	475.54	0	0	642.881	0	0
10		874.897	0.089	0.010	1110.061	0.709	0.063	626.529	0.048	0.076
11		1004.701	0.208	0.020	680.41	0.036	0.052	1128.636	1.133	0.100
12		1050.732	0.352	0.033	912.708	0.676	0.074	1166.03	3.317	0.284
13		1142.275	1.139	0.099	977.552	2.079	0.212	1076.513	4.503	0.418
14		1022.637	1.817	0.177	772.916	3.025	0.391	908.81	4.25	0.467
15		741.372	3.462	0.466	721.871	4.104	0.568	708.321	4.323	0.610
16		652.168	4.383	0.672	558.176	4.23	0.757	615.165	5.35	0.869
17		539.845	5.2	0.963	587.273	6.257	1.065	512.912	7.512	1.464

**Table 12:** Exergy analysis of developed setup with latent heat storage integration at 12 cm depth.

Time	Depth	SSSS			SSSS (LH)			SSSS (LH + SAH)		
		Ex_in_ (kJ/m^2^)	Ex_out_ (kJ/m^2^)	n_eff_ (%)	Ex_in_ (kJ/m^2^)	Ex_out_ (kJ/m^2^)	n_eff_ (%)	Ex_in_ (kJ/m^2^)	Ex_out_ (kJ/m^2^)	n_eff_ (%)
9	12 cm	822.006	0	0	690.05	0	0	279.808	0	0
10		875.929	0.101	0.011	966.637	0.2	0.020	996.648	0.282	0.028
11		1123.905	0.205	0.018	894.786	0.42	0.046	1036.16	0.775	0.074
12		1033.443	0.12	0.011	1165.322	0.779	0.066	1033.486	0.934	0.090
13		1097.023	1.702	0.155	1241.56	2.254	0.181	1051.656	2.185	0.207
14		1006.562	2.527	0.251	955.034	2.59	0.271	902.318	2.7	0.299
15		810.99	2.573	0.317	658.867	2.943	0.446	743.906	3.572	0.480
16		641.668	2.442	0.380	708.642	2.862	0.403	559.447	3.097	0.553
17		530.196	2.755	0.519	542.574	3.79	0.698	643.832	5.664	0.879

**Table 13:** Exergy analysis of developed setup with latent heat storage integration at 15 cm depth.

Time	Depth	SSSS			SSSS (LH)			SSSS (LH)+ SAH		
		Ex_in_ (kJ/m^2^)	Ex_out_ (kJ/m^2^)	n_eff_ (%)	Ex_in_ (kJ/m^2^)	Ex_out_ (kJ/m^2^)	n_eff_ (%)	Ex_in_ (kJ/m^2^)	Ex_out_ (kJ/m^2^)	n_eff_ (%)
9	15 cm	830.795	0	0	718.103	0	0	894.941	0	0
10		1033.495	0.014	0.013	843.562	0.176	0.020	1031.002	0.040	0.038
11		1134.622	0.165	0.014	959.687	0.215	0.022	1161.268	0.879	0.075
12		1042.921	0.505	0.048	1170.913	0.569	0.048	1169.928	3.233	0.276
13		1280.533	1.007	0.078	1200.362	0.97	0.080	1197.352	8.543	0.713
14		939.93	0.815	0.086	1170.608	2.367	0.202	1113.756	13.244	1.189
15		726.667	1.316	0.181	751.847	3.935	0.523	936.651	15.403	1.644
16		605.151	1.993	0.329	734.659	4.026	0.548	671.065	12.483	1.860
17		582.195	2.702	0.464	586.46	3.3	0.562	634.139	14.289	2.253

**Table 14:** Exergy analysis of developed setup with hybrid heat storage integration at 3 cm depth.

Time	Depth	SSSS			SSSS (SH+LH)			SSSS (SH+LH + SAH)		
		Ex_in_ (kJ/m^2^)	Ex_out_ (kJ/m^2^)	n_eff_ (%)	Ex_in_ (kJ/m^2^)	Ex_out_ (kJ/m^2^)	n_eff_ (%)	Ex_in_ (kJ/m^2^)	Ex_out_ (kJ/m^2^)	n_eff_ (%)
9	3 cm	915.75	0	0	802.065	0	0	861.567	0	0
10		1130	0.298	0.026	909.84	0.268	0.031	1094.373	0.765	0.073
11		1170.738	0.35	0.029	1046.931	0.410	0.041	1167.694	2.707	0.243
12		1221.475	1.24	0.101	1182.845	1.677	0.148	1211.038	5.154	0.446
13		1258.443	3.307	0.262	1196.586	6.737	0.591	1219.301	12.264	1.056
14		1002.189	4.793	0.478	986.691	10.404	1.107	1013.89	15.315	1.586
15		801.77	7.502	0.935	507.024	13.074	2.702	799.575	18.796	2.468
16		708.693	8.137	1.148	351.776	13.836	4.130	477.983	23.893	5.236
17		606.22	10.366	1.709	117.280	15.628	14.989	97.208	16.853	18.204

**Table 15:** Exergy analysis of developed setup with hybrid heat storage integration at 6 cm depth.

Time	Depth	SSSS			SSSS (SH+LH)			SSSS (SH+LH + SAH)		
		Ex_in_ (kJ/m^2^)	Ex_out_ (kJ/m^2^)	n_eff_ (%)	Ex_in_ (kJ/m^2^)	Ex_out_ (kJ/m^2^)	n_eff_ (%)	Ex_in_ (kJ/m^2^)	Ex_out_ (kJ/m^2^)	n_eff_ (%)
9	6 cm	915.626	0	0	734.126	0	0	851.500	0	0
10		726.528	0.173	0.023	1167.840	0.699	0.062	811.039	1.107	0.143
11		1086.761	0.427	0.039	796.766	0.500	0.065	1294.188	2.505	0.202
12		1132.074	1.432	0.126	1064.389	2.362	0.233	1298.904	3.664	0.296
13		1114.5	1.763	0.158	991.694	2.471	0.261	1311.889	5.305	0.424
14		946.445	3.5	0.369	943.022	3.748	0.417	1037.862	5.423	0.548
15		828.394	4.964	0.599	837.745	5.984	0.750	803.085	5.825	0.761
16		726.181	6.248	0.860	665.988	7.173	1.130	612.922	12.709	2.176
17		558.908	6.592	1.179	509.345	8.053	1.660	536.587	14.000	2.739

**Table 16:** Exergy analysis of developed setup with hybrid heat storage integration at 9 cm depth.

Time	Depth	SSSS			SSSS (SH+LH)			SSSS (SH+LH + SAH)		
		Ex_in_ (kJ/m^2^)	Ex_out_ (kJ/m^2^)	n_eff_ (%)	Ex_in_ (kJ/m^2^)	Ex_out_ (kJ/m^2^)	n_eff_ (%)	Ex_in_ (kJ/m^2^)	Ex_out_ (kJ/m^2^)	n_eff_ (%)
9	9 cm	905.227	0	0	499.317	0	0	675.025	0	0
10		874.897	0.089	0.010	1165.564	0.744	0.066	657.855	0.050	0.080
11		1004.701	0.208	0.020	714.431	0.037	0.055	1185.067	1.189	0.105
12		1050.732	0.352	0.033	958.343	0.709	0.077	1224.381	3.483	0.298
13		1142.275	1.139	0.099	1026.429	2.182	0.223	1130.339	4.728	0.439
14		1022.637	1.817	0.17	811.561	3.176	0.410	954.250	4.462	0.490
15		741.372	3.462	0.466	758.964	4.309	0.596	743.737	4.539	0.640
16		652.168	4.383	0.672	586.084	4.441	0.795	645.923	5.617	0.913
17		539.845	5.2	0.963	616.636	6.569	1.118	538.557	7.888	1.537

**Table 17:** Exergy analysis of developed setup with hybrid heat storage integration at 12 cm depth.

Time	Depth	SSSS			SSSS (SH+LH)			SSSS (SH+LH + SAH)		
		Ex_in_ (kJ/m^2^)	Ex_out_ (kJ/m^2^)	n_eff_ (%)	Ex_in_ (kJ/m^2^)	Ex_out_ (kJ/m^2^)	n_eff_ (%)	Ex_in_ (kJ/m^2^)	Ex_out_ (kJ/m^2^)	n_eff_ (%)
9	12 cm	822.006	0	0	724.552	0	0	293.798	0	0
10		875.929	0.101	0.011	1014.968	0.210	0.021	1046.480	0.296	0.029
11		1123.905	0.205	0.018	939.525	0.441	0.049	1088.968	0.813	0.078
12		1033.443	0.12	0.011	1228.588	0.817	0.070	1085.160	0.980	0.094
13		1097.023	1.702	0.155	1303.638	2.366	0.190	1104.239	2.294	0.218
14		1006.562	2.527	0.251	1002.785	2.719	0.284	947.434	2.835	0.314
15		810.99	2.573	0.317	691.809	3.090	0.469	781.101	3.750	0.504
16		641.668	2.442	0.380	744.074	3.005	0.424	587.419	3.251	0.581
17		530.196	2.755	0.519	569.702	3.978	0.733	676.023	5.947	0.923

**Table 18:** Exergy analysis of developed setup with hybrid heat storage integration at 15 cm depth.

Time	Depth	SSSS			SSSS (SH+LH)			SSSS (SH+LH + SAH)		
		Ex_in_ (kJ/m^2^)	Ex_out_ (kJ/m^2^)	n_eff_ (%)	Ex_in_ (kJ/m^2^)	Ex_out_ (kJ/m^2^)	n_eff_ (%)	Ex_in_ (kJ/m^2^)	Ex_out_ (kJ/m^2^)	n_eff_ (%)
9	15 cm	830.795	0	0	754.008	0	0	939.688	0	0
10		1033.495	0.014	0.013	885.740	0.184	0.0219	1082.552	0.042	0.040
11		1134.622	0.165	0.014	1007.671	0.225	0.0235	1219.331	0.922	0.079
12		1042.921	0.505	0.048	1229.458	0.597	0.0500	1238.423	3.394	0.290
13		1280.533	1.007	0.078	1260.380	1.018	0.0848	1257.219	8.970	0.749
14		939.930	0.815	0.086	1233.138	2.485	0.212	1169.444	13.906	1.248
15		726.667	1.316	0.181	789.439	4.131	0.549	983.483	16.173	1.726
16		605.151	1.993	0.329	771.392	4.227	0.575	704.618	13.107	1.953
17		582.195	2.702	0.464	615.783	3.465	0.590	665.846	15.003	2.365

### 5.2 Embodied energy analysis

#### 5.2.1 Single slope solar still

For the manufacturing of single slope solar still, the following materials were used as shown in the Table 19. Table 19 illustrates the distribution of the embodied energy of the materials used. The major percentage contributor of the embodied energy in this system are aluminium angle, metal frame and glass.

**Table 19:** Embodied analysis of single slope solar still.

S.no	Item	Wt	Energy	Total Energy
1	Glass	5.5	7.28	40.04
2	Putty	1	1.472	1.47
3	Metal sheet	2	55.28	110.56
4	Frame	20	5.55	111
(i)	1"×1mm section	3.59	55.28	198.45
(ii)	4"×1mm section	0.82	55.28	45.33
(iii)	1"×3mm angle	0.08	55.28	4.42
5	Fitting			
(i)	Hinges/*kabja*	0.2	55.28	11.06
(ii)	*Kundi(Door lock)*	0.025	55.28	1.38
(iii)	Handle	0.1	55.28	5.53
(iv)	steel screw	0.25	9.67	2.42
	**Embodied Energy**			**531.66 kWh**

The distribution of the total embodied energy used during fabrication is shared in the ratio of 46%, 21% and 13% respectively for aluminium angle, metal frame and glass. The embodied energy of of Single Slope Solar Still is 531.66 kWh.

#### 5.2.2 Single slope solar still with hybrid heat storage

For the manufacturing of Single Slope Solar Still with Hybrid Heat Storage the distribution of embodied energy of the used material is illustrated by Table 20. In the existing system, the major percentage contributors of the embodied energy are paraffin wax, aluminium angle and metal sheets.

**Table 20:** Embodied analysis of single slope solar still with hybrid heat storage.

S.no	Item	Wt	Energy	Total Energy
1	Glass	5.5	7.28	40.04
2	Putty	1	1.472	1.47
3	Metal sheet	2	55.28	110.56
4	Frame	20	5.55	111
(i)	1"×1mm section	3.59	55.28	198.45
(ii)	4"×1mm section	0.82	55.28	45.33
(iii)	1"×3mm angle	0.08	55.28	4.42
5	Fitting			
(i)	Hinges/*kabja*	0.2	55.28	11.06
(ii)	*Kundi(Door lock)*	0.025	55.28	1.38
(iii)	Handle	0.1	55.28	5.53
(iv)	steel screw	0.25	9.67	2.42
6	Paraffin wax	35	16	560
7	Black Gravel	29	0.04	1.16
8	Aluminium Jacket	2	55.28	110.56
	**Embodied Energy**			**1203.38 kWh**

Of the total embodied energy utilised during fabric, the sharing ratio was 38%, 17% and 13% respectively for paraffin wax, aluminium angle and metal sheets. Embodied Energy analysis of Single Slope Solar Still with Hybrid Heat Storage is found to be 1203.38 kWh.

#### 5.2.3 Single slope solar still with hybrid heat storage & solar air heater

For the manufacturing of Single Slope Solar Still with Hybrid Heat Storage & Solar Air Heater, the distribution of embodied energy of the used material is illustrated by Table 21. In the existing system, the major percentage contributors of the embodied energy are paraffin wax, aluminium angle and metal frame.

**Table 21:** Embodied analysis of single slope solar still with Hybrid heat storage & Solar air heater.

S.no	Item	Wt	Energy	Total Energy
1	Mirror	5.4	7.28	39.31
2	Coating	0.75	0.278	0.20
3	Black PVC sheet	0.325	19.44	6.32
4	Aluminium angle	4.815	55.28	
(i)	1"×1mm section	3.59	55.28	198.45
(ii)	4"×1mm section	0.82	55.28	45.33
(iii)	1"×3mm angle	0.08	55.28	4.42
5	Fitting			
(i)	Hinges/*kabja*	0.2	55.28	11.05
(ii)	*Kundi(Door lock)*	0.025	55.28	1.38
(iii)	Handle	0.1	55.28	5.52
(iv)	steel screw	0.25	9.67	2.42
6	Steel tray	0.7	9.67	6.77
7	Gravel	29	0.04	1.16
8	Aluminium Jacket	2	55.28	110.56
9	Thermocol	0.25	29.39	7.35
10	Wooden Frame	10.5	0.44	4.62
11	Glass	10	7.28	72.80
12	Putty	2	1.472	2.944
13	Metal sheet	3	55.28	165.84
14	Frame	20	5.55	111
15	Pipe	1	15.59	15.59
16	Paraffin wax	35	16	560
	**Embodied Energy**			**1373.03 kWh**

Of the total embodied energy utilised during fabric, the sharing ratio was 33%, 15% and 12% respectively for paraffin wax, aluminium angle and metal frame. Embodied Energy of Single Slope Solar Still with Hybrid Heat Storage & Solar Air Heater embodied energy is found to be 1373.03 kWh.

### 5.3 Energy payback time and cost analysis of the developed set up

The Energy Payback Time (EPBT) of the hybrid heat storage-based single slope solar still coupled with a solar air heater is observed to be higher compared to the single slope solar still. This is attributed to its intricate construction and operational complexities. The hybrid system utilizes thermal storage for the heat transfer and desalination processes.

Specifically, the EPBT for the hybrid heat storage-based single slope solar still coupled with a solar air heater is determined to be 1.87 years. In contrast, the EPBT values for the single slope solar still with hybrid heat storage and the conventional single slope solar still are found to be 1.65 years and 0.95 years, respectively.

The cost analysis of all solar still arrangements is carried out in this section to calculate the CPL of freshwater obtained from the still. Most of the materials required for the fabrication and development of different solar still arrangements are available in the local market at an affordable price. Some of the materials like ply board, glass wool and iron are readily and cheaply available from a scrap vendor. This decreases the overall initial cost of the system. Evacuated tubes in used form are also easily available in the markets of some big cities. In terms of cost analysis, the hybrid system incurs a cost of $234. In comparison, the single slope solar still with hybrid heat storage and the conventional single slope solar still have associated costs of $189 and $129, respectively. The detailed breakdown of costs is presented in Tables 22–24.

**Table 22:** Cost analysis of single slope solar still with Hybrid heat storage & solar air heater.

Item	Price ($)
Paraffin	50
Installation and testing	38
Metal supports	22
Glass cover	21
Water copper basin	19
Valves and pipes	14
Insulation material	11
Galvanized steel box	11
Reflectors	10
Black Gravel	10
Feed water tank	8
Fresh water tank	5
Wooden box	15
**TOTAL**	**234 $**

**Table 23:** Cost analysis of single slope solar still with hybrid heat storage.

Item	Price ($)
Paraffin	50
Installation and testing	30
Water copper basin	19
Insulation material	11
Galvanized steel box	11
Metal supports	15
Reflectors	10
Glass cover	10
Valves and pipes	10
Black Gravel	10
Feed water tank	8
Fresh water tank	5
**TOTAL**	**189 $**

**Table 24:** Cost analysis of single slope solar still.

Item	Price ($)
Installation and testing	30
Water copper basin	19
Metal supports	15
Insulation material	11
Galvanized steel box	11
Reflectors	10
Glass cover	10
Valves and pipes	10
Feed water tank	8
Fresh water tank	5
**TOTAL**	**129 $**

The investment payback period for the three solar still arrangements was calculated based on their per-day freshwater production rates. The Hybrid System produces 769 ml/m^2^h, the Single Slope Solar Still with Hybrid Heat Storage produces 644 ml/m^2^h, and the Conventional Single Slope Solar Still produces 425 ml/m^2^h. Assuming six hours of daily operation, the Hybrid System generates 4.614 liters of water per day, the Single Slope Solar Still with Hybrid Heat Storage produces 3.864 liters per day, and the Conventional Single Slope Solar Still produces 2.550 liters per day. Over the course of a year, this amounts to annual productions of 1684.11 liters, 1410.36 liters, and 930.75 liters, respectively. Using a water cost of $0.20 per liter, the annual savings for each system were calculated as $336.82 for the Hybrid System, $282.07 for the Single Slope Solar Still with Hybrid Heat Storage, and $186.15 for the Conventional Single Slope Solar Still.

The payback period for each system was determined by dividing the initial investment by the annual savings. For the Hybrid System, which has an initial cost of $234, the payback period is approximately 0.70 years. The Single Slope Solar Still with Hybrid Heat Storage, costing $189, has the shortest payback period at 0.67 years. The Conventional Single Slope Solar Still, with an initial cost of $129, has a payback period of around 0.69 years. All three systems offer a quick return on investment, typically within less than a year, with the Single Slope Solar Still with Hybrid Heat Storage showing the best performance in terms of payback efficiency.

### 5.4 CO_2_ emission and CO_2_ mitigation of developed solar still

To assess the adverse environmental impact of a hybrid heat storage-based single slope solar still coupled with a solar air heater, as well as a hybrid heat storage-based single slope solar still and a single slope solar still, it is crucial to calculate the annual CO_2_ emissions. The recorded CO_2_ emissions per year were 44.54 kg/year, 38.02 kg/year, and 33.87 kg/year for the hybrid heat storage-based single slope solar still coupled with a solar air heater, hybrid heat storage-based single slope solar still, and single slope solar still, respectively.

The net mitigation of CO_2_ was found to be higher when utilizing the hybrid heat storage-based single slope solar still coupled with a solar air heater. Specifically, the net mitigation of CO_2_ for the hybrid heat storage-based single slope solar still coupled with a solar air heater was 50.26 tonnes, whereas for the hybrid heat storage-based single slope solar still and the single slope solar still, it was 46.27 tonnes and 38.32 tonnes, respectively.

The earned carbon credits for desalination varied, with the hybrid heat storage-based single slope solar still coupled with a solar air heater ranging from 18344.21 to 73376.83 INR. For the hybrid heat storage-based single slope solar still and the single slope solar still, the carbon credits were found to be in the range of 16893.13 to 67572.53 INR and 13990.98 to 55963.94 INR, respectively.

## 6. Conclusions

The study emphasizes the importance of addressing exergy losses and environomical factors to enhance solar still efficiency, offering valuable insights for sustainable water desalination technology. The major conclusions are as follows:

- Energy efficiency decreased as water depth increased, from 35% at 3 cm depth to 31.72% at 15 cm depth. This highlights the need for optimizing water depth to enhance system performance.
- The incorporation of Sensible Heat (SH), Latent Heat (LH), and Hybrid Heat Storage (SH+LH) improved efficiencies by SSSS(SH): 37.5%, SSSS(LH): 38% and SSSS(SH+LH): 38.7% respectively.
- The integration of Solar Air Heaters (SAH) further increased these efficiencies, with the Hybrid system + SAH reaching the highest efficiency of 40.8%.
- The absorber plate contributed 86% of the total exergy destruction, with a maximum exergy destruction rate of 717 W/m^2^.
- Other components showed lower exergy destruction: 61.1 W/m^2^ for the glass cover and 50.2 W/m^2^ for saline water. Addressing these exergy losses is crucial for improving system efficiency.
- In the hybrid heat storage-based system, the major contributors to embodied energy were paraffin wax (38%), aluminum angles (17%), and metal frames (13%).
- The total embodied energy for the hybrid system was 1203.38 kWh, which increased to 1373.03 kWh when coupled with a Solar Air Heater.
- The investment payback period analysis reveals that the Single Slope Solar Still with Hybrid Heat Storage is the most efficient, with a payback period that is approximately 4.29% shorter than the Conventional Single Slope Solar Still and 4.29% shorter than the Hybrid System, highlighting a small but notable improvement in economic performance.
- The hybrid heat storage-based single slope solar still with a Solar Air Heater achieved the highest CO₂ mitigation at 50.26 tonnes, compared to 46.27 tonnes for the hybrid system and 38.32 tonnes for the conventional system.
- Earned carbon credits ranged from INR 18,344.21 to INR 73,376.83, depending on the system configuration.

The above findings have practical applications in the design and optimization of solar desalination systems, particularly for regions facing water scarcity. By incorporating hybrid heat storage systems and solar air heaters, solar stills can be made more energy-efficient, cost-effective, and environmentally sustainable, making them suitable for widespread use in off-grid areas and low-resource environments.

## 7. Limitations and future scope

The study has several limitations that open avenues for future research. One of the primary limitations is the restricted range of water depths tested, which were confined to between 3 cm and 15 cm. While useful, this range may not capture the full spectrum of optimal water depths for varying environmental conditions, suggesting the need for broader experimentation. Additionally, the study only considers two thermal energy storage materials—black gravel for sensible heat and paraffin wax for latent heat—without exploring other phase-change materials or alternative sensible heat storage options. Future research could expand on this by incorporating different materials to assess their potential for improved performance.

## Supporting information

S1 Data (ZIP)
